# Effect of Newcastle disease virus level of infection on embryonic length, embryonic death, and protein profile changes

**DOI:** 10.14202/vetworld.2018.1316-1320

**Published:** 2018-09-24

**Authors:** Dahliatul Qosimah, Sri Murwani, Edhy Sudjarwo, M. Arfan Lesmana

**Affiliations:** 1 Laboratory of Microbiology and Immunology, Faculty of Veterinary Medicine, Brawijaya University, Indonesia; 2 Department of Poultry Production, Faculty of Animal Husbandry, Brawijaya University, Indonesia; 3 Animal Clinic, Faculty of Veterinary Medicine, Brawijaya University, Indonesia

**Keywords:** embryo, Newcastle disease, pathological change, protein, titer, virus

## Abstract

**Background and Aim:**

Newcastle disease virus (NDV) is an obligate intracellular parasite. Virus can only live on living cells. The embryonated chicken eggs (ECEs) are one of the growth media of virus that is a cheap, easy to do, and accurate for showing patterns of virus change in the host. Higher virus titers indicate the higher number of viruses and more virulent to infect host. This research aimed to investigate the effect of different level of NDV titer infection in ECEs on protein profile, embryonic length, mortality, and pathological change.

**Materials and Methods:**

The study used a completely randomized design of six treatments and seven replications. The treatments were different level of NDV titer infection in allantoic fluid (AF) of 9-11 days ECEs, i.e., P1=2^0^, P2=2^6^, P3=2^7^, P4=2^8^, P5=2^9^, and P6=2^10^ hemagglutination unit (HAU). All samples were separated by sodium dodecyl sulfate-polyacrylamide gel electrophoresis. Data were analyzed using one-way ANOVA with p=0.05 for length of the embryo and descriptive analysis for embryo mortality, pathology change, and protein band.

**Results:**

The result showed that protein profile of NDV-infected ECEs of all different levels is more complex than protein profile of no NDV-infected ECEs. NDV infected of all different levels showed longer size embryo, higher mortality embryo at the first 2 days, and higher occurrence of hemorrhagic in all part of bodies of embryo than those of no NDV infected.

**Conclusion:**

It was concluded that NDV infection of all different level decreased health conditions of chicken embryo of ECEs of 9-11 days old. Different level of NDV infection of ECEs of 9-11 days old showed no significantly different embryo profiles. However, all of the NDV-infected embryos were shorter, death on the 2nd day, and suffered more hemorrhage on all body surfaces than uninfected NDV embryos.

## Introduction

Newcastle disease (ND) is caused by ND virus (NDV) which is a genus of *Avulavirus* and *Paramyxoviridae* family. The incidence of ND that attacks poultry shows manifestations of gastrointestinal, respiratory, and neurological disorders that cause deaths of up to 100% depending on the viral pathotype [1,2]. ND virus is highly contagious and causes huge economic losses in the poultry industry due to the decrease in production and quality of eggs and chicken performance [3]. Virulent of NDV strains are endemic to poultry in most of Asia, Africa, and some northern and southern states of America. This disease can also be transmitted to humans. ND disease first epidemic in Java Island, Indonesia in 1926 and spread pandemically in chickens and other poultry subsequently found in Newcastle upon Tyne, England in 1927 [4,5].

NDVs can invade the host through inhalation, fecal-oral transmission routes, excretion of infected poultry, and contact through virus-contaminated equipment [2]. Isolation of the ND virus can come from fecal and nasopharyngeal swabs, as well as organs. ND virus can be grown in embryonated chicken eggs (ECEs) and cultured chicken embryo fibroblast cells [6].

NDV virulence detection is important for vaccination and eradication programs [7]. This study aimed to evaluate the effect of administering various doses of NDV infection to pathogenicity cause death and organ disorders in ECEs and as well as protein profiles in allantoic fluid (AF) as the basic research reference for the development of biomolecular research.

## Materials and Methods

### Ethical approval

This research had received ethical approval from the Ethics Commission of Faculty of Medicine of Brawijaya University, Malang (Ethic Certificates No 803-KEP-UB).

### Research method

The study used a completely randomized design of six treatments and seven replications. The treatments were different level of NDV infection of 9-11 days ECEs, i.e., P1=2^0^, P2=2^6^, P3=2^7^, P4=2^8^, P5=2^9^, and P6=2^10^ hemagglutination unit (HAU). Protein profile, embryonic length, mortality, and pathological change were observed, and the data were descriptively analyzed and using analysis of variance.

### Propagation and harvest of NDV in ECEs

Propagation of NDV in ECEs of 9-11 days old is negative against NDV antibodies (based on HA and hemagglutination inhibition [HI] test that showed titer of antibody negative). Based on observation using polymerase chain reaction methods on AF of ECEs, it was identified that NDV had F protein with a nucleotide length of 362 bP. Furthermore, the ECEs were put in egg incubator at 37°C and observed daily for viral death through light observation. The optimum standard temperature for embryo growth is 37.5-37.8°C [8]. The dead embryo shows no movement and black color. Embryos that have not shown death until the 7^th^ day, the embryo is forced off by being placed in the refrigerator at a temperature of 4°C. Collection of allantoic fluid from eggs infected by a virus was then measured by viral titers using HA test and HI test [9,10]. The stock of ND viruses in AF was stored at −20°C until use [11].

### NDV identification using HA test

HA test was used to find out the virus that has hemagglutinin protein to agglutinate 1% chicken erythrocytes. The samples used were AF from viral infection yield to ECEs. HA titers exhibit the highest dilution indicating viral proteins that are still capable of agglutinating erythrocytes [12,13]. Samples were inserted in a 96-well microtiter plate with a “V” shape base. Test was done in duplo.

### NDV identification using HI Test

HI test was performed according to OIE [9] using viral antigen ND 4 HA unit per well using 1% erythrocyte chicken. HI titers show the highest dilution of specific antibodies that are capable of inhibiting viruses to agglutinate the virus expressed as log2. The test was repeated twice.

### Mean death time test

NDV with titer variation of 10^6^-10^10^ was infected on seven ECEs for each treatment subsequently incubated at 37°C. Embryonic death was observed daily for 7 days and evaluated through candling and recorded embryonic mortality. The highest dilution in which all embryos die was calculated as mean lethal dose [7]. Viral virulence was categorized by three classifications: Embryo mortality <60 h (Velogenic), 60-90 h (Mesogenic), and > 90 h (Lentogenic) [3].

### Sodium dodecyl sulfate-polyacrylamide gel electrophoresis (SDS-PAGE) test

Protein profile of AF of infected NDV embryo of ECEs was analyzed by SDS-PAGE test. A total of 5 μl of AF was introduced into 7.5% stacking gel and 12% separating gel, then electrophoresis was run at room temperature with a voltage of 200 V. After electrophoresis, the gel was stained using a 0.1% Coomassie blue solution for 24 h and destained using methanol and glacial acetic acid for approximately 3 h [14,15].

## Results

### Protein profile of AF from normal and NDV-infected embryo of ECEs

**Figure-1:**
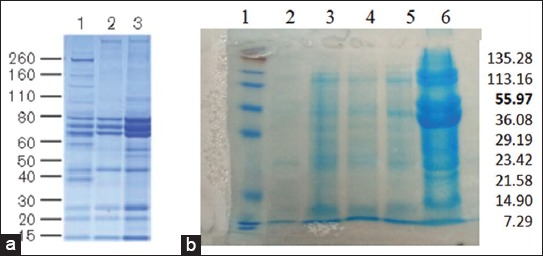
Protein profile of Newcastle disease virus (NDV) using sodium dodecyl sulfate-polyacrylamide gel electrophoresis (SDS-PAGE) test (Lane 1 left=molecular weight marker); (Lane 2=Titer virus 26), (Lane 3=Titer virus 27); (Lane 4=Titer virus 28); (Lane 5=Titer virus 29); (Lane 6=Titer virus 210). (a) Protein profile of allantoic fluid (AF) of no NDV-infected embryo as identified using SDS-PAGE, first lane [16]. (b) Protein profile of AF of NDV-infected embryo of ECEs 9-11 days of age.

Figure-1 presents the protein profile of AF of NDV-infected embryo of ECEs (B) as compared to those of AF from normal or no NDV-infected embryo of embryonated chicken eggs (B; [16]). All AFs of NDV-infected embryo of all different level showed same protein profile with a molecular weight ranged from 7.29 KDa to 135.28 KDa (Figure-1b), which were different from those AF of normal or no NDV-infected embryo (Figure-1a) [16].

### Influence of NDV titer level against embryo size

The results showed that level of NDV titer infection on ECEs 9-11 days of age did not show significantly different on embryo size but greater than those on non-infected NVD embryo (Table-1).

**Table-1:** Average chicken embryo size as affected by the level of NDV infection.

Treatment	Average±SD (cm)
No viral infection (2^0^)	3.96^a^
2^6^ HAU	5.26^b^
2^7^ HAU	4.92^b^
2^8^ HAU	5.26^b^
2^9^ HAU	4.88^b^
2^10^ HAU	5.08^b^

Values with different superscript in the same column showed significantly different (p<0.05), SD=Standard deviation. NDV=Newcastle disease virus

### Influence of NDV titer level against of embryonic pathology

**Figure-2:**
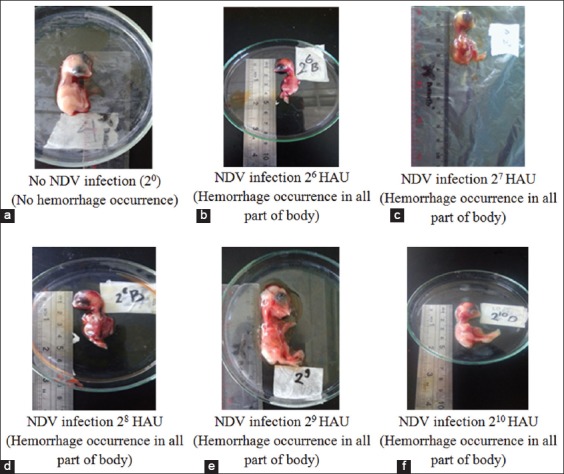
Gross pathology of the embryo in level infection of ND virus. (a) No virus infection (20) showed no hemorrhage in all part of the body; (b-f) inoculation with virus titer 2^6^, 2^7^, 2^8^, 2^9^, and 2^10^ hemagglutination unit showed hemorrhage in all part of the body.

Figure-2 showed gross pathology in all treatments. All NDV-infected embryos showed hemorrhage in all parts of the body, whereas no NDV-infected ones showed healthy embryo and no hemorrhage.

## Discussion

Protein profile of no NDV-infected chicken embryos is more complex than the protein profile of NDV-infected chicken embryos of all different levels. According to Dewi and Surachmi [17] that there are many serine protease-containing enzymes such as trypsin in the AF of ECEs that were infected with NDV. The enzymes degrade specific proteins on the molecular weight so that the gel will not absorb color and will be visualized as a band that is not stained. Trypsin can be found in the respiratory and digestive tract. Trypsin breaks down the F protein of NDV making it easier for NDV to replicate. Cleavage site sequence of the F protein of NDV, which increases the virulence of the NDV [18]. The protein profile of NDV-infected chicken embryos was clearly seen at infection level of 2^7^ HAU or higher.

The results of research are contrary to that done by Al-Garib *et al*. [19], which explains that NDV infection can lead to weight loss. NDV strains can replicate in the respiratory and digestive systems but preferably the digestive system. NDV can cause mass destruction of lymphoid organs in the gastrointestinal tract characterized by the presence of ulcers in the digestive epithelium. NDV could infect the digestive system causing proventriculus and intestinal hemorrhage and edema [20]. According to Kapczynski *et al*. [21] that pathological changes are associated with malabsorption syndrome in chickens as characterized by epithelial cell degeneration and crypta, also villus atrophy. The depth of the villi will increase in the infected embryo. Villi serve to absorb nutrients of food. Increased peristaltic movement of the intestines that decreased nutrient absorption and finally resulting in weight loss. NDV can also replicate in the respiratory system and lymphoid organs such as lung, spleen, fabricius, and thymus exchanges [6].

The ECEs used in this experiment did not contain antibodies against NDV because antibodies neutralized the virus and protect the embryo through two ways, first by binding infected cells, then reduce the production of progeny virus, and the second by releasing the progeny virus so that it will inhibit the spread of the virus [22].

The velogenic of NDV caused hemorrhage in all body systems. Hemorrhage was especially in the occipital part [7]. The infective dose of the ECEs was 2^3^, whereas the infective dose in the chicken was 2^6^ [23]. This was in accordance with Ahamed *et al*. [24] that the velogenic of NDV showed clinical symptoms of hemorrhage and necrosis of the respiratory tract and digestion. It also disrupted the nervous system [25]. Clinical symptoms appear varied depending on the strain of the virus, avian species and age, recurrent disease, and maternal immunity [2]. The results showed that all variations velogenic of NDV titer infected in ECEs caused the death of embryo on the 2^nd^ day. Death of embryos (100%) with a titer of NDV strains velogenic variation occurs within 48 h. This was consistent with Balachandran *et al*. [7] that NDV strains caused high mortality varies velogenic 60 h-90 h in ECEs infected with 2^5^-2^7^ NDV titers. This statement did not correspond to Mulisa *et al*. [26] that isolates velogenic NDV in birds caused death at 3-6 days post-infection. Immunity of embryo was strongly influenced by the immune system of the embryo, the amount of virus, and the virus strain.

The growth of the NDV titer 10^3.9^ TCID can be observed in Vero monolayer cells histopathologically showed the presence of cytopathic effect (CPE) followed by the formation of multinucleated giant cells (MGCs) 30-40 h after infection. CPE is characterized by granular cytoplasm around infected cells, development of microplaque, intracytoplasmic, vacuolization, and syncytia formation [27].

According to research conducted by Putra *et al*. [25], the embryos infected by the virus ND isolate Salatiga appear dark red and skin looks wet. Subcutaneous tissue containing blood and blood vessels stand out.

Embryonic mortality was influenced by several factors such as embryonic age, viral dosage, embryo’s immune status, incubation time, and environmental temperature. Infectious viruses can survive for months at room temperature in chicken eggs infected with the virus [8].

The NDV has an envelope, the single-stranded negative-sense RNA genome consists of six genes that encode the structural proteins and V and W proteins of the P gene called RNA editing [26].

Protein matrix (M), fusion (F) and hemagglutinin-neuraminidase (HN) of NDV related to the viral coat proteins which M function to perform virus budding formation, whereas F and HN protein mediate the entry and release of NDV. The virulence of NDV is determined by the amino acid composition of multiple basic amino acids from the breakdown of protein protease F [27]. F protein located on the virus envelope or outer sheath which mediates F of the virus with the host cell membrane. Protein F with host cells at neutral pH causes multinucleate (synticia) conditions that result in tissue necrosis and viral spread [5].

NDV protein profile showed molecular weight molecules tightly 7.29-135.28 Kda. This study contradicts Khan *et al*. [14] that the weight of NDV 12.5-181 Kda molecule uses 24-26 virus titers. During replication, NDV breaks down the glycoprotein precursors F0 to F1 and F2 for virus progeny to become infectious which can only occur in AF compared to culture cells. The NDV has six genes that affect viral pathogens, namely nucleoprotein, phosphoprotein (P), M, (F), HN, and polymerase (L). Virulence of the NDV ranges from avirulent (lentogenic), to mildly virulent (mesogenic) and highly virulent (velogenic), which can infect birds both naturally and experimentally [26].

Based on the results of the study showed that various variations of virulent titers of NDV (2^6^-2^10^) in ECEs showed death on the 2^nd^ day post-infection and the presence of hemorrhage but no significant difference to embryonic length than without infection. Recent studies have shown that a new protein profile is visible on the NDV 2^7^ titers.

## Conclusion

It was concluded that NDV infection of all different level decreased health conditions of chicken embryo of ECEs of 9-11 days old. Different level of NDV infection of ECEs of 9-11 days old showed no significantly different embryo profiles. However, all of the NDV-infected embryos were shorter, death on the 2^nd^ day, and suffered more hemorrhage on all body surfaces than uninfected NDV embryos.

## Authors’ Contributions

DQ was responsible for controlling the course of studies and analyzing data. SM did SDS-PAGE analysis. ES performed ND virus inoculation and harvested the results. MAL did HA and HI test. All authors read and approved the final manuscript.
